# Measure functional network and cortical excitability in post-anoxic patients with unresponsive wakefulness syndrome diagnosed by behavioral scales

**DOI:** 10.3389/fnins.2022.1071594

**Published:** 2023-01-11

**Authors:** Chen Li, Yong Wang, Wende Li, Yi Yang, Xiaoyu Xia

**Affiliations:** ^1^Department of Interventional and Vascular Neurosurgery, The Characteristic Medical Center of People’s Liberation Army (PLA) Rocket Force, Beijing, China; ^2^Zhuhai University of Macau (UM) Science & Technology Research Institute, Zhuhai, China; ^3^Senior Department of Neurosurgery, The First Medical Center of People’s Liberation Army (PLA) General Hospital, Beijing, China; ^4^Department of Neurosurgery, The Seventh Medical Center of People’s Liberation Army (PLA) General Hospital, Beijing, China; ^5^Department of Neurosurgery, Beijing Tiantan Hospital, Beijing, China

**Keywords:** functional connectivity, TMS-EEG, unresponsive wakefulness syndrome, global mean field amplitude, graph theory

## Abstract

**Background:**

Brain assessment shows great values in prognosis, treatment, resource allocation, and decision-making for patients with disorders of consciousness (DOC). However, less research focused on cortical conditions of patients with unresponsive wakefulness syndrome (UWS).

**Methods:**

We recorded resting-state EEG and TMS-EEG from post-anoxic patients with UWS, diagnosed by repeated Coma Recovery Scale-Revised (CRS-R). Measurements of functional connectivity and networks were performed by phase lock value (PLV) and network parameters of graph theory (average path length, clustering coefficient, and small-world). Global cortical reactivity values (GCRV) were used to assess cortical excitability.

**Results:**

The coefficient of variation (CV) presented marked inter-individual variations of PLV (*CV* = 0.285), network parameters (*CV* > 0.2), and GCRV (*CV* = 0.929) within these patients. The patients’ PLV and network parameters at theta and alpha bands significantly correlated with their GCRV values. Patients with higher PLV (*r* = 0.560, 0.406), as well as better preserved network (lower average path length (*r* = −0.522, −0.483), higher clustering coefficient (*r* = 0.522, 0.445), and small-world (*r* = 0.522, 0.445) at theta and alpha bands, presented higher GCRV. The functional connectivity, which is significantly correlated with frontal GCRV, is also mainly located in the frontal region. These correlations were not significant at other frequency bands: Delta, beta, and gamma bands.

**Conclusion:**

These findings suggested that the CRS-R-diagnosed post-anoxic patients with UWS had very different cortical conditions. Functional networks and cortical excitability measured by TMS-EEG could complement behavioral assessment to assess these patients’ cortical conditions.

**Significance:**

It provides a deeper understanding of neurophysiological dysfunction in patients with UWS and hints to the clinics that neural-electrophysiological assessment for such patients may be necessary to acquire their brain conditions, which may benefit stratified management for them.

## Introduction

Brain assessment is crucial for the treatment strategy making of patients with disorders of consciousness (DOC) (Kondziella et al., 2020). Patients with different cortical conditions showed differentiated responses to treatment (Thibaut et al., 2015; Naro et al., 2016). In clinics, behavioral scales are always used to evaluate the conscious states of patients with DOC, especially the Coma Recovery Scale-Revised (CRS-R) (Giacino et al., 2004). However, it is always limited by the injured body function of patients in their expression (Schnakers et al., 2009; Gosseries et al., 2016), as it is based on behavioral responses to external stimulation or commands. It reported that ∼40% of patients with DOC may be misdiagnosed based on CRS-R assessment (American Congress of Rehabilitation Medicine, Brain Injury-Interdisciplinary Special Interest Group, Disorders of Consciousness Task Force, Seel et al., 2010; Gosseries et al., 2016). Therefore, CRS-R is not an effective and direct approach to assess the brain conditions of patients with DOC.

Recently, multiple neural-electrophysiological technologies, such as electroencephalography (EEG) (Lehembre et al., 2012a; Bai et al., 2017b), concurrent transcranial magnetic stimulation and EEG (TMS-EEG) (Casali et al., 2013; Formaggio et al., 2016), and event-related potential (ERP) (Cruse et al., 2011, 2014), have been used to improve brain assessment of patients with DOC. Studies using EEG features in assessing the brain of patients with DOC are challenging and exciting. A large body of research suggested resting-state EEG could effectively evaluate cerebral cortex activity for the diagnosis, prognosis, and treatment effect in patients with DOC (Bai et al., 2017b). Among the complicated EEG characteristics, the measurement of functional connectivity and networks reflects the information interaction between distributed brain regions and conforms to the concepts of “integration” in the information integration theory, which was proposed to be the foundation of consciousness (Tononi, 2004; van Vugt et al., 2018). In general, the levels of functional connectivity of patients with DOC were found to be consistent with their levels of consciousness (Pollonini et al., 2010). This consistency was proven by the correlation between connectivity measures and clinical assessments of consciousness (Lehembre et al., 2012b). Patients with higher levels of consciousness showed higher power functional connectivity and better-connected networks than those with lower levels of consciousness (Lehembre et al., 2012b). In addition, EEG functional networks could predict metabolism and complement systematic behavioral assessment in DOC diagnosis (Chennu et al., 2017). Therefore, EEG functional networks would be considered convincing features in assessing neural-electrophysiological states of patients with DOC.

Transcranial magnetic stimulation-EEG, as an emerging technology, shows practical prospect in the assessment of the brain of patients with DOC (Bai et al., 2016). TMS-EEG can measure the interaction between various brain areas at the millisecond level, thus revealing information on subjects’ cortical excitability and reactivity (Sarasso et al., 2014). In patients with DOC, cortical excitability derived from TMS-EEG could effectively differentiate different consciousness states, for example, unresponsive wakefulness syndrome (UWS) formerly called vegetative state (VS), minimally conscious state (MCS), and locked-in syndrome (LIS) (Rosanova et al., 2012), and predict the consciousness recovery of patients with DOC (Bai et al., 2016). Patients with UWS show a simple, local cortical response to TMS, while patients with MCS and LIS have complex activations involving different brain areas and affecting large-scale cortex after TMS. Moreover, TMS-EEG could detect the immediate cortical responses to treatments, which would not be observed by clinical assessment (Bai et al., 2017a). Casali et al. (2013) quantified the cortical responses of TMS-EEG and proposed the perturbation complexity index to measure the level of consciousness. Several multi-modal studies also provided evidence verifying that the TMS-EEG characteristics correlate with the structural integrity (Bodart et al., 2018), metabolism (Bodart et al., 2017), and cortical injury (Gosseries et al., 2015) of the patients with DOC. Therefore, because of its high sensitivity to conscious alteration and close correlation with the fundamental brain conditions, TMS-EEG would be a critical technique to assess the brain conditions of patients with DOC.

A large number of studies addressed EEG functional networks and cortical excitability in patients with DOC. Less of them, however, focus on exploring the brain conditions of patients with UWS. Both traumatic and non-traumatic injuries result in patients with UWS. Non-traumatic injuries, especially anoxia, produce widespread damage to cortical and thalamic neurons. However, patients with UWS after anoxia do not invariably show diffuse neocortical neuronal loss (Schiff, 2010). We hypothesize that the patients with UWS after anoxia, especially the ones diagnosed by behavioral scales alone, would have divergent cortical conditions. Brain assessment would facilitate the inhomogenous management of such patients. Therefore, this study used EEG and TMS-EEG to investigate the functional networks of cortical excitability in post-anoxic patients with UWS diagnosed by CRS-R and to improve our knowledge of the cortical conditions of those patients.

## Materials and methods

### Patient

The clinical characteristics of patients are shown in Table 1. All participants had suffered severe anoxia and showed no severe cerebral atrophy by MRI scans. They had no epileptic history or EEG epileptiform activity, pacemakers, aneurysm clips, neurostimulators, or brain/subdural electrodes. All patients received routine medication and rehabilitation courses but no consciousness-influenced treatment in at least 2 months before this study, including zolpidem, modafinil, midazolam, and baclofen. None of the participants had suffered fever or infections 1 week before the EEG and TMS-EEG recording. Written informed consent to participate in this study was obtained from patients’ caregivers. This study was approved by the Ethics Committee of People’s Liberation Army (PLA) Army General Hospital.

**TABLE 1:** Demographic details for the patients.

Patient	Age	Sex	Cause	Months post-injury	CRS-R						
					**Auditory**	**Visual**	**Motor**	**Oro-motor**	**Comm**	**Arousal**	**Total**
P1	30	F	Acute myocardial	7	1	0	2	1	0	2	6
P2	60	M	Cardiac arrest	8	1	0	2	1	0	2	6
P3	50	F	Cardiopulmonary arrest	8	1	0	2	1	0	2	6
P4	35	M	Cardiac arrest	9	0	0	2	1	0	2	5
P5	43	M	Cardiac arrest	13	1	0	2	1	0	2	6
P6	42	F	Cardiopulmonary arrest	8	0	0	2	1	0	2	5
P7	52	F	Cardiac arrest	6	1	0	2	1	0	2	6
P8	70	F	Cardiac arrest	30	1	1	2	1	0	1	6
P9	62	M	Cardiopulmonary arrest	12	1	0	2	1	0	1	5
P10	23	F	Respiratory infarction	8	1	1	2	1	0	2	7
P11	26	M	Respiratory infarction	11	1	0	2	1	0	2	6
P12	34	F	Respiratory infarction	9	1	1	2	1	0	1	6
P13	56	M	Respiratory infarction	5	1	0	2	0	0	1	4
P14	28	M	Cardiac arrest	6	1	1	2	1	0	1	6
P15	44	M	Cardiopulmonary arrest	12	1	0	2	1	0	1	5

Comm, communication; CRS-R, Coma recovery scale-revised; F, female; M, male; UWS, unresponsive wakefulness syndrome.

### Behavioral assessment

Clinical assessment was carefully conducted by trained neurologists using repetitive Coma Recovery Scale-Revised (CRS-R) (Giacino et al., 2004). The CRS-R contains 23 items separated into six subscales (the visual, auditory, motor, and oromotor/verbal functions, communication, and arousal). Each patient received a minimum of three times of CRS-R assessment on the afternoon of a different day. The best result was kept as the behavioral diagnosis.

### Transcranial magnetic stimulation

A single TMS-EEG data acquisition session took 10–15 min. Each patient received 200 single pulses of TMS tangentially at the left dorsolateral prefrontal cortex (DLPFC) under navigation (navigate by Brainsight system and mark the target site at the electrode cap using a marking pen). DLPFC as a stimulating target is widely used in DOC research (Rosanova et al., 2012; Bai et al., 2016, 2017a). We used a Magstim *R*^2^ stimulator with a 70 mm figure-of-eight coil (Magstim Company Limited, Whitland, UK), which can produce a biphasic waveform with a pulse width of ∼0.1 ms. The TMS handle rotated posterior-laterally, approximately 45° to the middle line of the brain. Stimulation intensity for each patient was set as 120% of their resting motor threshold (RMT) (Ferreri et al., 2011). The RMT was defined as the lowest TMS intensity that can evoke at least 5 out of 10 EMG with an amplitude of > 50μ*V* peak to peak in the relaxed first dorsal interosseous muscle of the right hand. During the TMS-EEG recording, subjects were inserted earplugs, which continuously played a masking noise, to avoid TMS-evoked auditory potentials by the click associated with the TMS discharge. Bone conduction was attenuated by placing a thin layer of foam between the coil and scalp. Magnetic stimulation was administered in accordance with safety guidelines (Wassermann, 1998).

### Electroencephalography recordings and pre-processing

Transcranial magnetic stimulation-EEG and 20 min of resting-state EEG were recorded on the same day for all the patients, with resting-state EEG first and TMS-EEG followed. The signals were acquired by a TMS-compatible EEG recorder with 62 channels (BrainAmp 64 MRplus, Brain Products), with positions of the international 10–20 system. The equipment used TMS-compatible sintered Ag/AgCl-pin electrodes, with the skin/electrode impedance maintained below 5 kΩ. We set a band-pass filtered at DC to 1,000 Hz in the recorder, while EEG signals were digitized at a sampling rate of 2.5 kHz.

Offline analysis was performed using EEGLAB 12.0.2.5b, running in a MATLAB environment (version 2013b, MathWorks Inc., Natick, USA). For the TMS-EEG processing (1) EEG signals were segmented into epochs starting from 300 ms before to 500 ms after the TMS pulse onset (Massimini et al., 2005; Ferrarelli et al., 2010; Ferreri et al., 2011). (2) Data from 10 ms before to 20 ms after the TMS pulse were replaced using the cubic interpolation function of MATLAB (Thut et al., 2011) to exclude the TMS artifacts. (3) The 50 Hz power-line artifact was removed from the remaining trials using a notch filter. (4) EEG signals were down-sampled to 500 Hz and band-pass filtered (1–80 Hz). (5) Independent component analysis (ICA) was used to identify the evoked artifacts (such as eye movement, muscle artifacts, decay, and recharge artifacts), with visual inspection to assess scalp distribution, frequency, timing, and amplitude. The components deemed to be artifacts were removed using ICA (Casula et al., 2014). (6) Single trials were carefully inspected to remove residual TMS artifacts. (7) After the artifact reduction, at least 150 trials were preserved for each patient, and the baseline was corrected over 300 ms pre-stimulus. After processing, data were average-referenced; TMS-evoked EEG response was obtained by averaging over the trials.

For the resting-state EEG analysis (1) EEG signals were down-sampled to 500 Hz and band-pass filtered (1–45 Hz). (2) ICA was used to identify and remove the artifact-relevant components, such as eye movement and muscle activation. (3) The data were average-referenced and segmented into epochs of 10 s. Epochs with artifacts were removed by visual inspection.

### Functional connectivity

#### Phase-locking value

In the present study, functional connectivity was measured by PLV, which has been used in several previous studies (Rudrauf et al., 2006; Holz et al., 2010; Fell and Axmacher, 2011). We measured PLV in different frequency bands: full band (1–45 Hz), delta (1–4 Hz), theta (4–8 Hz), alpha (8–13 Hz), beta (13–30 Hz), and gamma (30–45 Hz). In this study, we give a brief description of the calculation. For the resting-state EEG epochs, we evaluated the instantaneous phase φ_*x*_(*t*) and φ_*y*_(*t*) of the pairwise channel, based on the Hilbert transform. Then, the phase difference was defined by as follows:


(1) $$\Delta \,\varphi_{x\,y}\,\left(t\right)=\varphi_{x}\,\left(t\right)-\varphi_{y}\,\left(t\right)$$

Several indices, based on the phase difference within the short term, can be used to indicate the phase synchronization between two series (Rosenblum et al., 2001). In this study, PLV based on the circular variance of the phase difference was applied as follows:


(2) $$P\,L\,V_{x\,y}=\frac{1}{N}\,\left|\sum_{t=1}^{N}e^{j\,\Delta \,\varphi_{x\,y}\,\left(t\right)}\right|$$

This measure of PLV varies from 0 to 1, and the computation involves no parameter choices. In this way, the functional connectivity can be described by phase synchronization matrix *C*, with each element of *PLV*_*xy*_.

Then, we used a surrogate method (the iterative amplitude-adjusted Fourier transform method) to correct the false coupling. In calculating the PLV between two channels, we randomly shuffled the phase of one signal and kept its spectrum unchanged. Then, a new surrogated PLV (*PLV*_*surro*_) can be obtained. After surrogating over N_*sum*_ times (N_*sum*_ > 50), PLV values greater than 95% statistical threshold (mean plus 1.96 times the standard deviation) of *PLV*_*surro*_ will be preserved, and the other is set to zero.

#### Graph theoretical analysis

To further describe the functional connectivity, graph theoretical analysis was performed based on the PLV matrix. The nodes in the graph were represented by the electrodes, while the links were defined by the measures of association between the nodes in the study’s PLV values.

Graphs can be characterized by various measures; in this study, synchronization matrix *C* was used to create weighted graphs. Average path length represented the average number of edges of the shortest path between the pairs of vertices. The clustering coefficient denoted the likelihood that neighbors of a vertex would also be connected to each other. Full definitions for the calculation of the clustering index (*C_w_*) and path length (*L_w_*) for the analysis of weighted networks have previously been described by Stam et al. (2009). To calculate the clustering index from weighted networks, the weights between node *i* and other nodes *j* should be symmetrical (ω_*ij*_ = ω_*ji*_) and, 0≤ω_*ij*_≤1 as proposed by Onnela et al. (2005). Indeed, both conditions are readily fulfilled when using PLV values as a weight definition. The weighted clustering index of vertex *i* was then defined as follows:


(3) $$C_{i}=\frac{\sum_{k\neq i}\sum_{l\neq i,l\neq k}\omega_{i\,k}\,\omega_{i\,l}\,\omega_{k\,l}}{\sum_{k\neq i}\sum_{l\neq i,l\neq k}\omega_{i\,k}\,\omega_{i\,l}}$$

Note that in all sums, terms with *k* = *i*, *l* = *i*, or *k* = *l* were skipped. The mean clustering of the total network was defined as follows:


(4) $$C_{w}=\frac{1}{N}\,\sum_{i=1}^{N}C_{i}$$

The length of a weighted path between two vertices was then defined as the sum of the lengths of the edges of this path. The shortest path *L*_*ij*_ between two vertices *i* and j was the path between *i* and j with the shortest length. The averaged path length of the entire network was computed as follows:


(5) $$L_{w}=\frac{1}{\left(1/N\,\left(N-1\right)\right)\,\sum_{i=1}^{N}\sum_{j\neq i}^{N}\left(1/L_{i\,j}\right)}$$

In the aforementioned formula, the harmonic mean was used to handle disconnected edges resulting in infinite path lengths, that is, 1/∞→0 (Newman, 2003). The small-world was then calculated as $\text{S}=\frac{C_{w}}{L_{w}}$.

### Cortical excitability

#### Global mean field power

To measure the TMS-evoked global response, a GMFP was used to describe the TMS-evoked potential (TEP). The GMFP can be expressed as follows:


(6) $$G\,M\,F\,P\,\left(t\right)=\sqrt{\sum_{i=1}^{N}{\left[V_{i}\,\left(t\right)-\bar{V}\,\left(t\right)\right]}^{2}/N}$$

where *V*_*i*_(*t*) is the signal averaged over trials measured on EEG channel *i* at time *t*, $\bar{V}\,\left(t\right)$ is the signal averaged over trials and channels at time *t*, and *N* is the number of channels. The GMFP identifies the maximum amplitude of the evoked field and is used to index the effect of TMS on global brain activities (Komssi et al., 2004). At each time of TEP peaks, we performed source modeling to investigate TMS-evoked cortical activation. Brainstorm software (Tadel et al., 2011)^1^ was used to compute the cortex, skull, and scalp meshes and co-register these meshes with EEG sensor positions by rigid rotations and translations of anatomical landmarks (nasion, left tragus, and right tragus). Conductive head volume was modeled according to the 3-spheres BERG method. The inverse solution was calculated on TEP by using the weighted minimum norm constraint.

#### Global cortical reactivity value

A global cortical reactivity value was measured to quantify the cortical responses to TMS pulses. First, a bootstrap method was used to shuffle 1,000 times the time samples of pre-stimulus activity (from −300 to −10 ms) of GMFP time series at a single-trial level. The maximum value across all latencies was selected at each shuffling, and the maximum distribution was used to assess a threshold for determining the significance of GMFP with a significance level of *p* < 0.01 (McCubbin et al., 2008; Gosseries et al., 2015). Then, the significant voltage values in post-stimulus (20–500 ms) of each GMFP time series were cumulated as the global index of cortical reactivity (Rosanova et al., 2009).

### Statistics

Inter-individual variations of the features were assessed by the coefficient of variation (CV) (ratio of the standard deviation to the mean). Correlational analyses of the functional network features (average PLV, average path length, cluster coefficient, and small-world) with the GCRV were measured using Kendall’s tau coefficient. *P* < 0.05 is the threshold for significance.

## Results

### Functional network and cortical excitability

**FIGURE 1:**
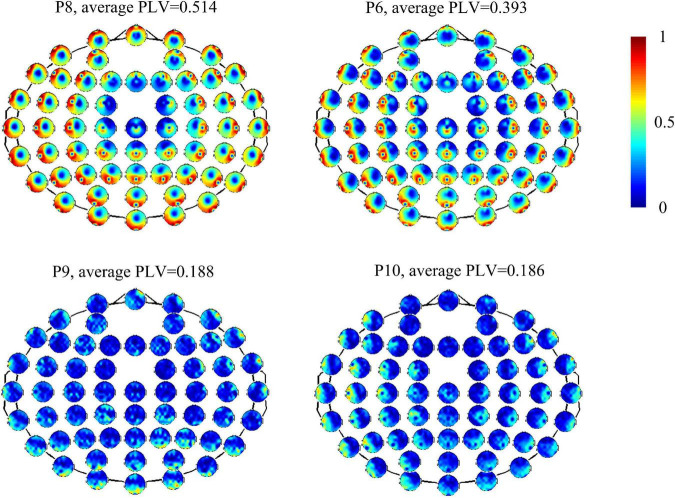
Functional connectivity measured by PLV of electrodes with overall brain in four patients (P8, P6, P9, and P10).

Figure 1 shows functional connectivity measured by PLV of four patients at the full band. It also shows different strengths and patterns of connectivity within patients. Patients 8 and 6 showed marked and strong global connectivity (P8: average *PLV* = 0.514, standard deviation = 0.229; P6: average *PLV* = 0.393, standard deviation = 0.237). Patients 9 and 10 had a relatively weak connectivity pattern (average *PLV* = 0.186 and standard deviation = 0.142). For all the patients, CV is 0.285 (maximum value = 0.514, minimum value = 0.186, standard deviation = 0.097, and mean = 0.340).

**FIGURE 2:**
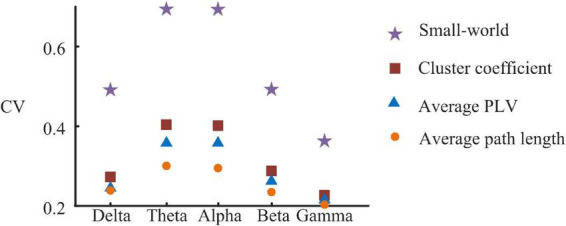
Relative coefficient of variation of functional network parameters.

To further describe the functional network, we calculated PLV and graphical network parameters at each frequency band. Table 2 gives mean values and standard variance values of the features at each frequency band. All the CV of the features are greater than 0.2 (Figure 2). Among them, small-world has the highest CV at each band, followed by the cluster coefficient.

**TABLE 2:** Average PLV and graphical network parameters (mean value and standard variance) at each frequency band.

	Delta	Theta	Alpha	Beta	Gamma
Average PLV	(0.367, 0.089)	(0.359, 0.127)	(0.342, 0.121)	(0.306, 0.079)	(0.285, 0.061)
Average path length	(3.028, 0.722)	(3.178, 0.950)	(3.316, 0.979)	(3.519, 0.823)	(3.663, 0.748)
Cluster coefficient	(0.334, 0.091)	(0.325, 0.131)	(0.307, 0.123)	(0.265, 0.076)	(0.243, 0.055)
Small-world	(0.121, 0.059)	(0.124, 0.086)	(0.111, 0.077)	(0.083, 0.041)	(0.071, 0.026)

**FIGURE 3:**
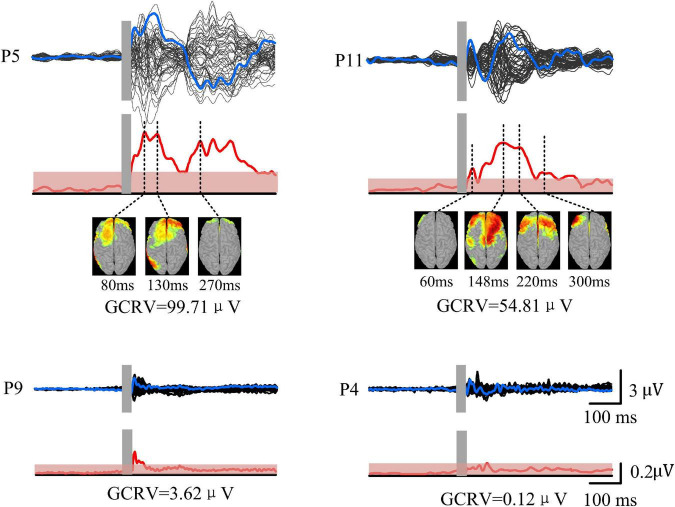
TMS-EEG of four patients (P5, P11, P9, and P4). Black curves show butterfly plots of TEP at all electrodes. Blue curves show TEP at F3 electrode (near target site). GMFP (red curves) was calculated based on the TEP. Red shadows show threshold values for determining significance of GMFP with significance level at *p* < 0.01. Source model was performed at each peak of TEP.

TMS-evoked potential and corresponding GMFP were measured for all patients (Figure 3). Figure 3 gives four samples of patients (P5, P11, P9, and P4). It showed that the patients had very different cortical responses to TMS in both temporal and spatial domains. P5 and P11 show distinct evoked components and marked response power upper threshold, but the evoked patterns are different between P5 and P11. In temporal, the evoked components in TEP of P11 mainly appear within 300 ms following TMS. However, P5 has significant evoked peaks after 300 ms. In addition, P5 shows more complex evoked components and stronger evoked power (*GCRV* = 99.71μV) than P11 (*GCRV* = 54.81μV). On the contrary, P9 and P4 show less evoked components and low evoked power (P9: *GCRV* = 3.62μV; P4: *GCRV* = 0.12μV), as shown at the bottom of Figure 1. This distinct inter-individual difference could be found within all the 15 patients: *CV* = 0.929 (maximum value = 107.007, minimum value = 0.120, standard deviation = 41.284, and mean = 44.440).

### Correlation between the functional network and the cortical excitability

**FIGURE 4:**
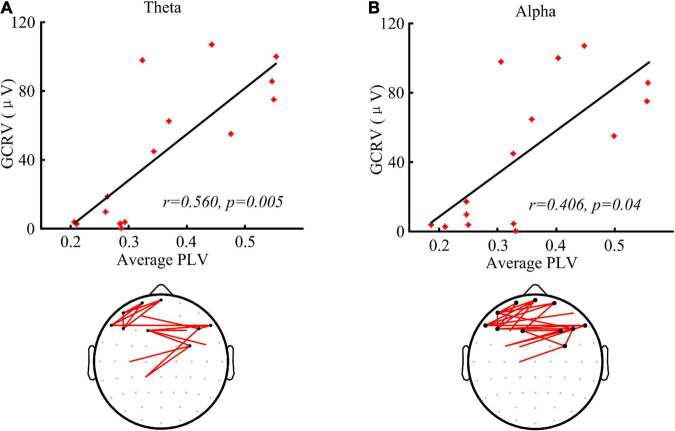
Functional connectivity measured by PLV and the correlation with the global cortical reactivity values (GCRV). **(A,B)** Correlation (Kendall’s tau coefficient) of global averaged PLV at theta **(A)** and alpha **(B)** band with the patients’ GCRV. Bottom panel shows the connectivity (red lines) that significantly correlate with the patients’ GCRV. Black dots show electrodes associated with at least three red lines.

As shown in Figure 4, the patients’ GCRV had a significantly positive correlation with the average PLV of the global brain at theta (*r* = 0.560, *p* = 0.005) and alpha (*r* = 0.406, *p* = 0.04) bands. The patients with higher average PLV in functional connectivity measurement showed higher GCRV in TMS-EEG measurement. In addition, the connectivity, which significantly correlated with the GCRV, either at theta or alpha bands (bottom panel of Figure 4) was mainly located at the frontal region. There was no significant correlation between the average PLV and the patients’ GCRV at other frequency bands.

**FIGURE 5:**
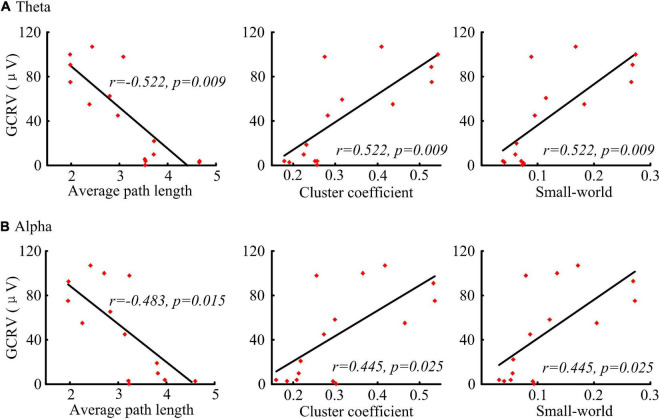
Correlation (Kendall’s tau coefficient) of the graphical network parameters with the patients’ GCRV. **(A)** Correlation of the average path length, cluster coefficient, and small-world at theta band with the patients’ GCRV. **(B)** Correlation of the average path length, cluster coefficient, and small-world at alpha band with the patients’ GCRV.

Significant correlations were found between the network parameters and the patients’ GCRV (Figure 5). Patients’ GCRV had a negative correlation with the average path length (theta: *r* = −0.522, *p* = 0.009; alpha: *r* = −0.483, *p* = 0.015) and a positive correlation with the cluster coefficient (theta: *r* = 0.522, *p* = 0.009; alpha: *r* = 0.445, *p* = 0.025) and the small-world (theta: *r* = 0.522, *p* = 0.009 alpha: *r* = 0.445, *p* = 0.025) at theta and alpha bands. Table 3 shows that there was no significant correlation between the GCRV with network features at other frequency bands.

**TABLE 3:** Correlation (Kendall’s tau coefficient) of functional network features with the patients’ global cortical reactivity values at delta, beta, and gamma bands.

	Delta	Beta	Gamma
Average PLV	*r* = 0.309 *p* = 0.218	*r* = 0.382, *p* = 0.121	*r* = 0.236, *p* = 0.359
Average path length	*r* = −0.309, *p* = 0.218	*r* = −0.418, *p* = 0.087	*r* = −0.200, *p* = 0.445
Cluster coefficient	*r* = 0.319, *p* = 0.308	*r* = 0.309, *p* = 0.218	*r* = 0.164, *p* = 0.542
Small-world	*r* = 0.319, *p* = 0.308	*r* = 0.309, *p* = 0.218	*r* = 0.164, *p* = 0.542

There was no significant correlation between the GCRV or functional network parameters with their clinical assessment (CRS-R), the patients’ age, and months post-injury.

## Discussion

The present study measured functional network features and cortical excitability features in post-anoxic patients with UWS. Big inter-individual variations of functional connectivity and cortical excitability were found in these patients. Some patients had markedly cortical responses to TMS and strong connectivity, whereas some patients showed relatively weak connectivity and low cortical excitability. In addition, the patients’ GCRV showed a significant correlation with their functional connectivity and network parameters at theta and alpha bands. The correlation did not exist at other frequency bands, such as delta, beta, and gamma bands.

The functional connectivity can represent common inputs from other brain areas, while the GCRV depends explicitly on the influence that one neural system exerts over another at the population level (Friston, 2011). In addition, a good network showed more efficient configuration (small average path length, high cluster coefficient, and high small-world) within the brain regions: Each small cortical region was more willing to connect to its neighbors, and it took them fewer steps to communicate with each other. These features all reflect the “integration” of the patients’ brains. They revealed very different cortical conditions among the patients in the present study. Furthermore, the residual brain network of patients with DOC correlated with their residual brain metabolism. A strong association has been demonstrated between functional connectivity and glucose metabolism (Chennu et al., 2017). TMS-EEG can measure cortical reactivity directly, and TEP presents the excitation changes in cortical circuits on a millisecond time scale (Ferreri et al., 2011; Bai et al., 2016). TMS-EEG study showed TMS triggered a simple local excitation change in patients with UWS, but TMS evoked MCS patients’ local and large-scale cortical responses (Rosanova et al., 2012). In summary, TMS-EEG measures proved a clear-cut difference in cortical excitability between patients with UWS and MCS. In addition, TMS-EEG also showed congruent results with glucose metabolism in patients with DOC (Bodart et al., 2017). Therefore, for patients with DOC, the residual functional network and preserved cortical excitability may share a similar physiological basis: cortical metabolism. To some extent, these findings provide a possible explanation for the correlation results in the patients, and patients who preserved better metabolism showed better cortical excitability and functional networks. On the contrary, studies of neuromodulation also support the findings: The responses of DOC patients’ brains to external stimulation depend on the residual brain network (Cavinato et al., 2015; Thibaut et al., 2015).

The patients we addressed in the present study are rare in clinics, as most of them tend to die within the first 2 years after injury. Previous studies always tend to research the brain conditions or consciousness states among patients with various etiologies or just with a rough classification: TBI and non-TBI. Less of them focused on homogenous groups such as post-anoxic patients with UWS. In behavioral assessed post-anoxic patients with UWS, studies reported that no significant EEG responses were elicited by TMS, even when TMS was delivered at high intensity at multiple stimulation sites (Ragazzoni et al., 2013; Gosseries et al., 2015), but these studies only included a few cases. Nevertheless, in such patients, different cortical responses to stimulation were also reported in ERP research. In three behavioral assessed post-anoxic patients with UWS, one patient showed a distinct N1 component, while the other two showed no meaningful evoked component (Ragazzoni et al., 2013). The present study is the first to focus on the brain conditions within such homogenous patients with UWS. Consistent with our hypothesis, the inter-individual variations denote the very different cortical conditions within the patients. It was the first time found that some behavioral assessed post-anoxic patients with UWS preserved good cortical excitability. On the contrary, the strength of functional connectivity and cortical excitability is generally consistent with conscious levels of patients with DOC (Casali et al., 2013; Sitt et al., 2014). Especially, the functional connectivity measured by phase coupling at theta and alpha bands was demonstrated as an efficient feature for capturing consciousness levels of patients with DOC (Lehembre et al., 2012b). However, these findings were not the case in the patients with UWS of the present study: The functional networks showed no correlation with patients’ CRS-R. Of course, considering the high rate of misdiagnosis when using CRS-R alone, there are enough reasons to speculate that the patients with better cortical excitability and functional networks may be actually in a better conscious state. Anyway, the findings of the present study revealed a necessity of depth assessment with the assistant of EEG or TMS-EEG. Acquiring more details about the brain conditions of the patients with UWS would raise diagnostic accuracy or improve stratified management of them in clinics.

Further study is needed to validate the findings of the present study. In the present study, the frontal region was selected as a target for detecting cortical excitability, as we considered that the frontal region is a crucial hub participating in the consciousness-related networks (Tononi, 2004, 2008; Alkire et al., 2008; Schiff, 2010). Frontal excitability would be very minded by non-invasive neuromodulation research (Angelakis et al., 2014; Thibaut et al., 2014; Naro et al., 2015; Cavaliere et al., 2016). In a way, it may lead to the results of frontal-located connectivity, which significantly correlates with GCRV. However, cortical excitability at other regions is still needed to validate the findings. Meanwhile, the analysis was conducted on UWS patients with the same cause (anoxia). More samples with various etiologies should be included to investigate whether the big inter-individual variations and correlations are ubiquitous in patients with UWS. Finally, future research should consider the relationship between the differences in cortical activity and the prognosis of post-anoxic patients with UWS.

## Conclusion

This is the first study which revealed that the post-anoxic patients with UWS, who were diagnosed by repeated CRS-R alone, had marked inter-individual variations of residual EEG networks and cortical excitability. The functional connectivity and cortical excitability showed significant correlations in those patients. These findings suggest us to measure functional networks and cortical excitability as complement assessments for such patients. It proved great values of neural-electrophysiological tools in assessing the brain conditions of patients with DOC.

## Data availability statement

The original contributions presented in this study are included in the article/supplementary material, further inquiries can be directed to the corresponding author.

## Ethics statement

The studies involving human participants were reviewed and approved by the Ethics Committee of PLA Army General Hospital. The patients/participants provided their written informed consent to participate in this study. Written informed consent was obtained from the individual(s) for the publication of any potentially identifiable images or data included in this article.

## Author contributions

CL and XX had full access to all the data in the study and takes responsibility for the integrity of the data. XX designed and conducted the study. YW and CL prepared the manuscript draft with important intellectual input from YY. YY and WL helped conduct the study, provided input, and helped with patient recruitment and consent. All authors listed have made a substantial, direct, and intellectual contribution to the work, and approved it for publication.

## Footnotes

1
http://neuroimage.usc.edu/brainstorm

## References

[B1] Alkire M. T. Hudetz A. G. Tononi G. (2008). Consciousness and anesthesia. *Science* 322 876–880. 10.1126/science.1149213 18988836 PMC2743249

[B2] American Congress of Rehabilitation Medicine, Brain Injury-Interdisciplinary Special Interest Group, Disorders of Consciousness Task Force, Seel R. Sherer M. Whyte J. Katz D. Giacino J. (2010). Assessment scales for disorders of consciousness. Evidence-based recommendations for clinical practice and research. *Arch. Phys. Med. Rehabil.* 91 1795–1813. 10.1016/j.apmr.2010.07.218 21112421

[B3] Angelakis E. Liouta E. Andreadis N. Korfias S. Ktonas P. Stranjalis G. (2014). Transcranial direct current stimulation effects in disorders of consciousness. *Arch. Phys. Med. Rehabil.* 95 283–289. 10.1016/j.apmr.2013.09.002 24035769

[B4] Bai Y. Xia X. Li X. (2017b). A review of resting-state electroencephalography analysis in disorders of consciousness. *Front. Neurol.* 8:471. 10.3389/fneur.2017.00471 28955295 PMC5601979

[B5] Bai Y. Xia X. Kang J. Yang Y. He J. Li X. (2017a). TDCS modulates cortical excitability in patients with disorders of consciousness. *Neuroimage Clin.* 15 702–709. 10.1016/j.nicl.2017.01.025 28702347 PMC5487253

[B6] Bai Y. Xia X. Kang J. Yin X. Yang Y. He J. (2016). Evaluating the effect of repetitive transcranial magnetic stimulation on disorders of consciousness by using TMS-EEG. *Front. Neurosci.* 10:473. 10.3389/fnins.2016.00473 27812319 PMC5071327

[B7] Bodart O. Amico E. Gómez F. Casali A. G. Wannez S. Heine L. (2018). Global structural integrity and effective connectivity in patients with disorders of consciousness. *Brain Stimul.* 11:358. 10.1016/j.brs.2017.11.006 29162503

[B8] Bodart O. Gosseries O. Wannez S. Thibaut A. Annen J. Boly M. (2017). Measures of metabolism and complexity in the brain of patients with disorders of consciousness. *Neuroimage Clin.* 14:354. 10.1016/j.nicl.2017.02.002 28239544 PMC5318348

[B9] Casali A. Gosseries O. Rosanova M. Boly M. Sarasso S. Casali K. (2013). A theoretically based index of consciousness independent of sensory processing and behavior. *Sci. Transl. Med.* 5:198ra105. 10.1126/scitranslmed.3006294 23946194

[B10] Casula E. P. Tarantino V. Basso D. Arcara G. Marino G. Toffolo G. M. (2014). Low-frequency rTMS inhibitory effects in the primary motor cortex: Insights from TMS-evoked potentials. *Neuroimage* 98 225–232. 10.1016/j.neuroimage.2014.04.065 24793831

[B11] Cavaliere C. Aiello M. Perri C. D. Amico E. Martial C. Thibaut A. (2016). Functional connectivity substrates for tDCS response in minimally conscious state patients. *Front. Cell. Neurosci.* 10:257. 10.3389/fncel.2016.00257 27857682 PMC5093112

[B12] Cavinato M. Genna C. Manganotti P. Formaggio E. Storti S. F. Campostrini S. (2015). Coherence and consciousness: Study of fronto-parietal gamma synchrony in patients with disorders of consciousness. *Brain Topogr.* 28 570–579. 10.1007/s10548-014-0383-5 25070585

[B13] Chennu S. Annen J. Wannez S. Thibaut A. Chatelle C. Cassol H. (2017). Brain networks predict metabolism, diagnosis and prognosis at the bedside in disorders of consciousness. *Brain* 140 2120–2132. 10.1093/brain/awx163 28666351

[B14] Cruse D. Beukema S. Chennu S. Malins J. G. Owen A. M. McRae K. (2014). The reliability of the N400 in single subjects: Implications for patients with disorders of consciousness. *Neuroimage Clin.* 4 788–799. 10.1016/j.nicl.2014.05.001 24936429 PMC4055893

[B15] Cruse D. Chennu S. Chatelle C. Bekinschtein T. A. Fernández-Espejo D. Pickard J. D. (2011). Bedside detection of awareness in the vegetative state: A cohort study. *Lancet* 378 2088–2094. 22078855 10.1016/S0140-6736(11)61224-5

[B16] Fell J. Axmacher N. (2011). The role of phase synchronization in memory processes. *Nat. Rev. Neurosci.* 12 105–118. 10.1038/nrn2979 21248789

[B17] Ferrarelli F. Massimini M. Sarasso S. Casali A. Riedner B. A. Angelini G. (2010). Breakdown in cortical effective connectivity during midazolam-induced loss of consciousness. *Proc. Natl. Acad. Sci. U.S.A.* 107 2681–2686. 20133802 10.1073/pnas.0913008107 PMC2823915

[B18] Ferreri F. Pasqualetti P. Määttä S. Ponzo D. Ferrarelli F. Tononi G. (2011). Human brain connectivity during single and paired pulse transcranial magnetic stimulation. *Neuroimage* 54 90–102. 10.1016/j.neuroimage.2010.07.056 20682352

[B19] Formaggio E. Cavinato M. Storti S. F. Tonin P. Piccione F. Manganotti P. (2016). Assessment of event-related EEG power after single-pulse TMS in unresponsive wakefulness syndrome and minimally conscious state patients. *Brain Topogr.* 29 322–333. 10.1007/s10548-015-0461-3 26590568

[B20] Friston K. J. (2011). Functional and effective connectivity: A review. *Brain Connect.* 1 13–36. 22432952 10.1089/brain.2011.0008

[B21] Giacino J. T. Kalmar K. Whyte J. (2004). The JFK coma recovery scale-revised: Measurement characteristics and diagnostic utility. *Arch. Phys. Med. Rehabil.* 85 2020–2029. 10.1016/j.apmr.2004.02.033 15605342

[B22] Gosseries O. Pistoia F. Charland-Verville V. Carolei A. Sacco S. Laureys S. (2016). The role of neuroimaging techniques in establishing diagnosis, prognosis and therapy in disorders of consciousness. *Open Neuroimag J.* 10 52–68. 10.2174/1874440001610010052 27347265 PMC4894918

[B23] Gosseries O. Sarasso S. Casarotto S. Boly M. Schnakers C. Napolitani M. (2015). On the cerebral origin of EEG responses to TMS: Insights from severe cortical lesions. *Brain Stimul.* 8 142–149. 10.1016/j.brs.2014.10.008 25481074

[B24] Holz E. M. Glennon M. Prendergast K. Sauseng P. (2010). Theta-gamma phase synchronization during memory matching in visual working memory. *Neuroimage* 52 326–335. 10.1016/j.neuroimage.2010.04.003 20382239

[B25] Komssi S. Kahkonen S. Ilmoniemi R. J. (2004). The effect of stimulus intensity on brain responses evoked by transcranial magnetic stimulation. *Hum. Brain Mapp.* 21 154–164. 10.1002/hbm.10159 14755835 PMC6871924

[B26] Kondziella D. Bender A. Diserens K. Erp W. V. Estraneo A. Formisano R. (2020). European academy of neurology guideline on the diagnosis of coma and other disorders of consciousness. *Eur. J. Neurol.* 27 741–756. 10.1111/ene.14151 32090418

[B27] Lehembre R. Gosseries O. Lugo Z. Jedidi Z. Chatelle C. Sadzot B. (2012a). Electrophysiological investigations of brain function in coma, vegetative and minimally conscious patients. *Arch. Ital. De Biol.* 150 122–139. 10.4449/aib.v150i2.1374 23165873

[B28] Lehembre R. Marie-Aurélie B. Vanhaudenhuyse A. Chatelle C. Cologan V. Leclercq Y. (2012b). Resting-state EEG study of comatose patients: A connectivity and frequency analysis to find differences between vegetative and minimally conscious states. *Funct. Neurol.* 27 41–47. 22687166 PMC3812750

[B29] Massimini M. Ferrarelli F. Huber R. Esser S. K. Singh H. Tononi G. (2005). Breakdown of cortical effective connectivity during sleep. *Science* 309 2228–2232. 10.1126/science.1117256 16195466

[B30] McCubbin J. Yee T. Vrba J. Robinson S. E. Murphy P. Eswaran H. (2008). Bootstrap significance of low SNR evoked response. *J. Neurosci. Methods* 168 265–272. 10.1016/j.jneumeth.2007.10.003 18054084 PMC2324207

[B31] Naro A. Bramanti P. Leo A. Russo M. Calabr R. S. (2016). Transcranial alternating current stimulation in patients with chronic disorder of consciousness: A possible way to cut the diagnostic gordian knot? *Brain Topogr.* 29 623–644. 10.1007/s10548-016-0489-z 27062669

[B32] Naro A. Russo M. Leo A. Bramanti P. Quartarone A. Calabr R. S. (2015). A single session of repetitive transcranial magnetic stimulation over the dorsolateral prefrontal cortex in patients with unresponsive wakefulness syndrome: Preliminary results. *Neurorehabil. Neural. Repair.* 29 603–613. 10.1177/1545968314562114 25539781

[B33] Newman M. E. (2003). Properties of highly clustered networks. *Phys. Rev. E Stat. Nonlin. Soft. Matter. Phys.* 68:026121. 10.1103/PhysRevE.68.026121 14525063

[B34] Onnela J. Saramäki J. Kertész J. Kaski K. (2005). Intensity and coherence of motifs in weighted complex networks. *Phys. Rev. E Stat. Nonlin. Soft. Matter. Phys.* 71:065103. 10.1103/PhysRevE.71.065103 16089800

[B35] Pollonini L. Pophale S. Situ N. Wu M. Frye R. E. Leon-Carrion J. (2010). Information communication networks in severe traumatic brain injury. *Brain Topogr.* 23 221–226. 20224956 10.1007/s10548-010-0139-9

[B36] Ragazzoni A. Pirulli C. Veniero D. Feurra M. Cincotta M. Giovannelli F. (2013). Vegetative versus minimally conscious states: A study using TMS-EEG, sensory and event-related potentials. *PLoS One* 8:e57069. 10.1371/journal.pone.0057069 23460826 PMC3584112

[B37] Rosanova M. Casali A. Bellina V. Resta F. Mariotti M. Massimini M. (2009). Natural frequencies of human corticothalamic circuits. *J. Neurosci.* 29 7679–7685. 19535579 10.1523/JNEUROSCI.0445-09.2009 PMC6665626

[B38] Rosanova M. Gosseries O. Casarotto S. Boly M. Casali A. Bruno M. (2012). Recovery of cortical effective connectivity and recovery of consciousness in vegetative patients. *Brain* 135 1308–1320. 22226806 10.1093/brain/awr340 PMC3326248

[B39] Rosenblum M. Pikovsky A. Kurths J. Schäfer C. Tass P. (2001). Phase synchronization: From theory to data analysis. *Handb. Biol. Phys.* 4 93–94.

[B40] Rudrauf D. Douiri A. Kovach C. Lachaux J. Cosmelli D. Chavez M. (2006). Frequency flows and the time-frequency dynamics of multivariate phase synchronization in brain signals. *Neuroimage* 31 209–227. 10.1016/j.neuroimage.2005.11.021 16413209

[B41] Sarasso S. Rosanova M. Casali A. G. Casarotto S. Fecchio M. Boly M. (2014). Quantifying cortical EEG responses to TMS in (un)consciousness. *Clin. Eeg Neurosci.* 45 40–49. 10.1177/1550059413513723 24403317

[B42] Schiff N. D. (2010). Recovery of consciousness after brain injury: A mesocircuit hypothesis. *Trends Neurosci.* 33 1–9. 10.1016/j.tins.2009.11.002 19954851 PMC2931585

[B43] Schnakers C. Vanhaudenhuyse A. Giacino J. Ventura M. Boly M. Majerus S. (2009). Diagnostic accuracy of the vegetative and minimally conscious state: Clinical consensus versus standardized neurobehavioral assessment. *BMC Neurol.* 9:35. 10.1186/1471-2377-9-35 19622138 PMC2718857

[B44] Sitt J. D. King J. Karoui I. E. Rohaut B. Faugeras F. Gramfort A. (2014). Large scale screening of neural signatures of consciousness in patients in a vegetative or minimally conscious state. *Brain* 137 2258–2270. 10.1093/brain/awu141 24919971 PMC4610185

[B45] Stam C. J. Haan W d Daffertshofer A. Jones B. F. Manshanden I. Walsum A. M. (2009). Graph theoretical analysis of magnetoencephalographic functional connectivity in Alzheimer’s disease. *Brain* 132 213–224. 10.1093/brain/awn262 18952674

[B46] Tadel F. Baillet S. Mosher J. C. Pantazis D. Leahy R. M. (2011). Brainstorm: A user-friendly application for MEG/EEG analysis. *Comput. Intell. Neurosci.* 2011:879716. 10.1155/2011/879716 21584256 PMC3090754

[B47] Thibaut A. Bruno M. Ledoux D. Demertzi A. Laureys S. (2014). tDCS in patients with disorders of consciousness: Sham-controlled randomized double-blind study. *Neurology* 82 1112–1118. 10.1212/WNL.0000000000000260 24574549

[B48] Thibaut A. Perri C. D. Chatelle C. Bruno M. Bahri M. A. Wannez S. (2015). Clinical response to tDCS depends on residual brain metabolism and grey matter integrity in patients with minimally conscious state. *Brain Stimul.* 8 1116–1123. 10.1016/j.brs.2015.07.024 26471400

[B49] Thut G. Veniero D. Romei V. Miniussi C. Schyns P. Gross J. (2011). Rhythmic TMS causes local entrainment of natural oscillatory signatures. *Curr. Biol.* 21 1176–1185. 10.1016/j.cub.2011.05.049 21723129 PMC3176892

[B50] Tononi G. (2004). An information integration theory of consciousness. *BMC Neurosci.* 5:42. 10.1186/1471-2202-5-42 15522121 PMC543470

[B51] Tononi G. (2008). Consciousness as integrated information: A provisional manifesto. *Biol Bull.* 215 216–242. 10.2307/25470707 19098144

[B52] van Vugt B. Dagnino B. Vartak D. Safaai H. Panzeri S. Dehaene S. (2018). The threshold for conscious report: Signal loss and response bias in visual and frontal cortex. *Science* 360 537–542. 10.1126/science.aar7186 29567809

[B53] Wassermann E. M. (1998). Risk and safety of repetitive transcranial magnetic stimulation: Report and suggested guidelines from the international workshop on the safety of repetitive transcranial magnetic stimulation, June 5–7, 1996. *Electroencephalogr. Clin. Neurophysiol.* 108 1–16. 10.1016/s0168-5597(97)00096-8 9474057

